# Diagnostic error rates and associated factors for lower gastrointestinal perforation

**DOI:** 10.1038/s41598-021-04762-y

**Published:** 2022-01-19

**Authors:** Taku Harada, Takashi Watari, Satoshi Watanuki, Juichi Hiroshige, Seiko Kushiro, Taiju Miyagami, Syunsuke Syusa, Satoshi Suzuki, Tetsuya Hiyoshi, Suguru Hasegawa, Shigeki Nabeshima, Hidetoshi Aihara, Shun Yamashita, Masaki Tago, Fumitaka Yoshimura, Kotaro Kunitomo, Takahiro Tsuji, Masanori Hirose, Tomoya Tsuchida, Taro Shimizu

**Affiliations:** 1 Division of General Medicine, Showa University Koto Toyosu Hospital, 5-1-38 Toyosu Koto-ku, Tokyo, 135-8577 Japan; 2 Department of Diagnostic and Generalist Medicine, Dokkyo Medical University Hospital, Mibu, Tochigi Japan; 3 General Medicine Center, Shimane University Hospital, Enya-cho, Japan; 4 Division of Emergency and General Medicine, Tokyo Metropolitan Tama Medical Center, Fuchu, Japan; 5 Department of General Medicine, Faculty of Medicine, Juntendo University, Tokyo, Japan; 6 Department of General Medicine, Tone Chuo Hospital, Numata, Gunma Japan; 7 General Medicine Department, Faculty of Medicine, Fukuoka University, Fukuoka, Japan; 8 Department of Gastroenterological Surgery, Faculty of Medicine, Fukuoka University, Fukuoka, Japan; 9 Department of General Medicine, Saga University Hospital, Saga, Japan; 10 Department of General Medicine, Yushima Clinic, Kumamoto, Japan; 11 Department of General Medicine, Kumamoto Medical Center, Kumamoto, Japan; 12 Division of General Internal Medicine, Department of Internal Medicine, St. Marianna University School of Medicine, Kanagawa, Japan

**Keywords:** Gastroenterology, Medical research

## Abstract

Lower gastrointestinal perforation is rare and challenging to diagnose in patients presenting with an acute abdomen. However, no study has examined the frequency and associated factors of diagnostic errors related to lower gastrointestinal perforation. This large-scale multicenter retrospective study investigated the frequency of diagnostic errors and identified the associated factors. Factors at the level of the patient, symptoms, situation, and physician were included in the analysis. Data were collected from nine institutions, between January 1, 2015 and December 31, 2019. Timely diagnosis was defined as diagnosis at the first visit in computed tomography (CT)-capable facilities or referral to an appropriate medical institution immediately following the first visit to a non-CT-capable facility. Cases not meeting this definition were defined as diagnostic errors that resulted in delayed diagnosis. Of the 439 cases of lower gastrointestinal perforation identified, delayed diagnosis occurred in 138 cases (31.4%). Multivariate logistic regression analysis revealed a significant association between examination by a non-generalist and delayed diagnosis. Other factors showing a tendency with delayed diagnosis included presence of fever, absence of abdominal tenderness, and unavailability of urgent radiology reports. Initial misdiagnoses were mainly gastroenteritis, constipation, and small bowel obstruction. In conclusion, diagnostic errors occurred in about one-third of patients with a lower gastrointestinal perforation.

## Introduction

Recently, there has been a remarkable increase in the level of attention and development in the field of diagnostic errors, with the volume of publications having rapidly increased^1^. The frequency and impact of diagnostic errors on medical care are increasing, with diagnostic errors reported in 5% of outpatient cases^2^, 10% of in-hospital deaths^3^, and 7–17% of in-hospital adverse events^4,5^. In the area of medical litigation, diagnostic errors are also reportedly the most common complaint^6^. A report by the National Academy of Medicine has identified diagnostic errors as an urgent national concern^7^ and the Economic Cycle Research Institute (ECRI) has designated diagnostic errors as the number one patient-safety problem. To prevent harm associated with diagnostic errors, it is first necessary to determine their frequency and identify their associated factors^8^.

Lower gastrointestinal perforation is an acute abdominal condition in which numerous bacteria from the stool spread from the small and large intestines into the abdominal cavity, causing acute diffuse peritonitis. Compared to upper gastrointestinal perforation, lower gastrointestinal perforation is less common but more severe, and is on the rise^9^. Regardless of the cause, the clinical presentation of small and large bowel perforations is relatively consistent, and management comprises resuscitation, antimicrobial therapy, and repair or reconstruction of the perforation site^10^. Although rare, it is common to experience difficulty in diagnosing lower gastrointestinal perforation, in timely fashion, in clinical practice. The preoperative diagnostic rate is approximately 10% with a high mortality rate at 30%^11^. Thus, lower gastrointestinal perforation is an important and lethal condition in clinical practice. However, to the best of our knowledge, no previous studies have examined the frequency of diagnostic errors in cases of lower gastrointestinal perforation and the factors associated with these diagnostic errors. To address this important clinical issue, we conducted a large-scale retrospective study to determine the frequency of diagnostic errors and to identify related factors.

## Methods

This retrospective study was conducted across nine institutions, between January 1, 2015 and December 31, 2019. Medical records were reviewed and data were extracted for all possible cases of lower gastrointestinal perforation, during the relevant period, using the following inclusion criteria: newly registered lower gastrointestinal perforation in the electronic medical record and use of the terms "free air" and "perforation" in the CT report. After review of the medical records of identified cases, the following cases were retained: cases in which clinical symptoms consistent with a lower gastrointestinal tract perforation and CT reports by a radiologist indicating lower gastrointestinal tract perforation; and cases in which a lower gastrointestinal tract perforation was confirmed by surgery, endoscopy, or contrast study. Excluded were cases with a diagnosis of perforated appendix, diverticulitis, and perforation of the upper gastrointestinal. The following case were also excluded: cases where surgery was not indicated and the perforation site, either in the upper or lower gastrointestinal tract, could not be determined by clinical symptoms or imaging findings; cases in which the site of perforation could not be identified even though surgery was performed due to free air on CT; gastrointestinal perforation due to endoscopy or after surgery; gastrointestinal perforation due to trauma; secondary perforation due to mesenteric ischemia; and patient < 15 years of age. For hospitals where electronic medical records were introduced during the study period, only cases evaluated following the introduction of the electronic medical records were included.

Data collected retrospectively included patient factors (age, gender, facility resident, diabetes, dementia, psychiatric disorders, chronic laxative use, chronic analgesic use, chronic antipsychotic use, use of immunosuppressive agents [including steroids], last-minute enemas, and bedridden status), disease factors (presence of fever, abdominal pain, acute pain, abdominal tenderness, elevated C-reactive protein [CRP], and foreign body), situational factors (outpatient or inpatient, time from onset to clinical or hospital visit, facility size [greater than or less than 400 beds], teaching hospital [with or without residency programs], and availability of urgent radiology reports), characteristics of the examining physician (general practitioner, emergency physician, gastroenterologist, or other), site of perforation (the small bowel, cecum, ascending colon, transverse colon, descending colon, sigmoid colon, and rectum), and whether the perforation was caused by a foreign body or not. Due to the characteristics of the department, the general practitioners and emergency physicians were defined as generalists.

### Definition of timely diagnosis, delayed diagnosis, and diagnostic error

We have determined two sets of criteria for timely diagnosis. The first of these patterns is a diagnosis of perforation of the lower gastrointestinal tract during the initial examination at a CT-capable facility. The second is a diagnosis of perforation of the lower gastrointestinal tract after initial examination at a non-CT facility with immediate referral to an appropriate medical institution for investigation of an acute abdomen or abdominal pain. Patients who had an incorrect diagnosis made during the initial examination at a non-CT facility but who were immediately referred to an appropriate medical institution for further investigation, resulting in a timely diagnosis, were defined as a “near miss.” Patients who had no evidence of perforation on initial CT but who were finally diagnosed with lower gastrointestinal perforation after additional evaluation, were defined “no fault” cases. Delayed diagnosis, therefore, included all cases without a timely diagnosis, as well as near miss and no fault cases.

### Analysis methods

The chi-squared test or the Fisher’s exact test was used to compare nominal variables. For continuous variables, *t*-tests or Wilcoxon rank-sum tests were used, as appropriate. For multiple logistic analysis, we incorporated several important factors that were likely to be significant (at *P* < 0.1) and that avoided multicollinearity, as follows: elderly patients (> 65 years old), visit to the health care facility after 24 h, presence of fever, absence of abdominal tenderness, elevated CRP (above 10 mg/L), night shift, first visit to a teaching hospital, unavailability of urgent radiology reports, first visit to the clinic, and examination by a non-generalist. Statistical analyses were performed using the EZR (Easy R) software^12^. All the tests were two-sided, with *P* < 0.05 considered statistically significant.

### Ethics statement

This study was approved by the ethical review board of Showa University Koto Toyosu Hospital (No.20T7044) and conducted in accordance with the Declaration of Helsinki. Written informed consent was waived owing to the retrospective study design by the ethical review board of Showa University Koto Toyosu Hospital.

## Results

Of the 439 cases with lower gastrointestinal perforation that were identified in the nine hospitals included, 205 (46.8%) were female and the median age was 72.21 ± 13.92 years. Considering the perforation sites, 230 were determined by surgery, 205 by CT, 2 by endoscopy, and 2 by contrast. Of the 439 cases, delayed diagnosis, near miss, and no fault occurred in 138 (31.4%), 34 (7.7%), and 17 (3.9%) cases, respectively. The near-miss and no-fault cases were grouped into the timely diagnosis group. The distribution of the patient, disease, situational, and physician factors according to the two groups, namely the “Timely” and “Delayed” diagnosis groups, is shown in Table 1. Significant differences were noted between the two groups in terms of visits within 6 h (odds ratio [OR]: 0.47, 95% confidence interval [CI]: 027–0.80), visits following more than 24 h (OR: 1.578, 95% CI: 1.037–2.403), presence of fever (OR: 1.648, 95% CI: 1.078–2.520), absence of abdominal tenderness (OR: 2.761, 95% CI: 1.457–5.232), elevated C-reactive protein (CRP) (OR: 2.820, 95% CI: 1.438–5.523), night shift (OR: 0.560, 95% CI: 0.345–0.909), visit to a teaching hospital (OR: 0.355, 95% CI: 0.234–0.537), first visit to the clinic (OR: 4.061, 95% CI: 2.455–6.717), unavailability of urgent radiology reports (OR: 2.306, 95% CI: 1.390–3.823), and examination by a non-generalist (OR: 5.882, 95% CI: 2.796–12.356).

**Table 1:** Comparison of the characteristics for participants and factors associated with the diagnosis.

Characteristics	Timely diagnosis (n = 301), frequency (%)	Delayed diagnosis (n = 138), frequency (%)	Odds ratio [95%CI ]	*P* value
**Patient factor**				
Age, meadn (SD),y	73.1 ± 13.2	70.3 ± 15.3		*P* = 0.047
Female, n (%)	139 (46.2)	66 (47.8)	1.165 [0.7786–1.744]	*P* = 0.758
Facility resident	29 (9.6)	7 (5.1)	0.501 [0.219–1.151]	*P* = 0.134
Diabetes	48 (15.9)	15 (10.9)	0.643 [0.349–1.185]	*P* = 0.188
Dementia	32 (10.6)	10 (7.2)	0.657 [0.318–1.36]	*P* = 0.298
Bedridden	13 (4.3)	9 (6.5)	1.546 [0.659–3.627]	*P* = 0.250
Psychiatric disorders	14 (4.7)	10 (7.2)	1.602 [0.707–3.632]	*P* = 0.266
Chronic analgesic use	40 (13.3)	20 (14.5)	1.106 [0.623 -1.964]	*P* = 0.765
Chronic antipsychotic use	20 (6.6)	12 (8.7)	1.338 [0.643–2.785]	*P* = 0.435
Chronic laxative use	74 (24.6)	33 (23.9)	0.964 [0.603–1.541]	*P* = 0.905
Use of immunosuppressive(including steroids)	43 (14.3)	16 (11.6)	0.787 [0.429–1.443]	*P* = 0.547
Last-minute enemas	13 (4.3)	4 (2.9)	0.661 [0.223–1.967]	*P* = 0.600
Age ≧ 65	23 4(77.7)	100 (72.5)	0.753 [0.476–1.193]	*P* = 0.231
**Time for visit**				
Within 6 h	80 (26.6)	20 (14.5)	0.468 [0.275–0.799]	*P* = 0.005
6–24 h	58 (19.3)	21 (15.2)	0.752 [0.438–1.292]	*P* = 0.350
More than 24 h	89 (29.6)	55 (39.9)	1.578 [1.037–2.403]	*P* = 0.038
Unclear	74 (24.6)	42 (30.4)	1.342 [0.859–2.096]	*P* = 0.202
**Disease factor**				
Presence of fever	132 (47.5)	76 (59.8)	1.648 [1.078–2.520]	*P* = 0.024
Abdomonal pain	260 (90.3)	117 (90.0)	0.969 [0.489–1.917]	*P* = 1.000
Acute pain	165 (72.7)	58 (65.9)	0.726 [0.429–1.229]	*P* = 0.270
Absence of abdominal tenderness	20 (7.1)	22 (17.5)	2.761 [1.457–5.232]	*P* = 0.002
Elevated C-reactive protein( above 10 mg/L)	198 (72.8)	83 (88.3)	2.820 [1.438–5.523]	*P* = 0.002
Perforation by a foreign body	16 (5.3)	8 (6.3)	1.188 [0.507–2.788]	*P* = 0.654
**Site of perfration**				
Rectum	28 (9.3)	13 (9.4)	1.014 [0.514–2.004]	*P* = 1.000
Sigmoid colon	140 (46.5)	60 (43.5)	0.885 [0.590–1.326]	*P* = 0.606
Descending colon	18 (18.6)	8 (5.8)	0.968 [0.419–2.237]	*P* = 1.000
Transverse colon	10 (3.3)	1 (0.7)	0.212 [0.035–1.305]	*P* = 0.185
Ascending colon	19 (6.3)	6 (4.3)	0.675 [0.271–1.682]	*P* = 0.509
Cecum	4 (1.3)	5 (3.6)	2.791 [0.798–9.758]	*P* = 0.147
Small bowel	73 (24.3)	43 (31.2)	1.414 [0.906–2.206]	*P* = 0.131
Difficult to identify the detailed area	9 (3.0)	2 (1.4)	0.477 [0.115–1.991]	*P* = 0.515
**Situational factor**				
Night shift	89 (38.4)	31 (25.8)	0.560 [0.345–0.909]	*P* = 0.024
First visit at teaching hospital	194 (64.5)	54 (39.1)	0.355 [0.234–0.537]	*P* < 0.001
First visit at large hospital(more than 400 beds)	115 (38.2)	26 (18.8)	0.375 [0.232–0.609]	*P* < 0.001
First visit at small hospital(less than 400 beds)	153 (50.8)	66 (47.8)	0.887 [0.593–1.326]	*P* = 0.608
First visit at clinic	33 (11.0)	46 (33.3)	4.061 [2.455–6.717]	*P* < 0.001
Unavailability of urgent radiology reports	206 (68.4)	115 (83.3)	2.306 [1.390–3.823]	*P* = 0.001
Clinic or hospital	43 (14.3)	46 (33.3)	3.000 [1.862–4.834]	*P* < 0.001
Gastroenterologist's consultation at first visit	64 (21.3)	35 (25.4)	1.258 [0.786–2.014]	*P* = 0.389
Emergency phycisian's consulation at first visit	53 (17.6)	5 (3.6)	0.176 [0.071–0.438]	*P* < 0.001
General phycisian's consulation at first visit	27 (9.0)	3 (2.2)	0.226 [0.072–0.713]	*P* = 0.007
Other physician's consultation af first visit	106 (35.2)	56 (40.6)	1.256 [0.831–1.899]	*P* = 0.288
Unknown's consulation at first visit	51 (17.0)	39 (28.3)	1.931 [1.201–3.107]	*P* = 0.008
Examined by a non-generalist(other than general phycisian and emergency physician)	221 (73.4)	130 (94.2)	5.882 [2.796–12.356]	*P* < 0.001

The results of the multivariate logistic regression analysis performed using the above-mentioned nine variables are shown in Table 2. We hypothesized at the beginning of the study that delayed diagnosis was more likely to occur in the elderly. Regarding age, we conducted a multivariate analysis using age ≥ 65 years as one of the variables. Using this age cutoff, examination by a non-generalist was significantly associated with a greater likelihood of delayed diagnosis (OR: 3.46, 95% CI: 1.13–10.60). Other factors with a tendency to be associated with a delayed diagnosis included: presence of fever (OR: 2.09, 95% CI: 0.90–4.83), absence of abdominal tenderness (OR: 3.27, 95% CI: 0.90–11.90), and unavailability of urgent radiology reports (OR: 3.15, 95% CI: 0.97–10.20).

**Table 2:** Results of the multivariate logistic regression analysis on associated factors of lower gastrointestinal perforation.

Multivariate logistic regression analysis	Odds ratio [95%CI]	*P* value
Elderly (> 65 years old)	0.79 [0.32–1.92]	0.597
Time for visit after 24 h	1.05 [0.40–2.74]	0.928
Presence of fever	2.09 [0.90–4.83]	0.086
Absence of abdominal tenderness	3.27 [0.90–11.90]	0.071
Elevated C-reactive protein(above 10 mg/L)	1.29 [0.44–3.78]	0.642
Night shift	1.16 [0.47–2.91]	0.747
First visit at teaching hospital	1.23 [0.46–3.25]	0.679
Unavailability of urgent radiology reports	3.15 [0.97–10.20]	0.056
First visit at clinic	2.45 [0.71–8.42]	0.156
Examined by a non-generalist	3.46 [1.13–10.60]	0.029

Of the 138 cases with delayed diagnosis, the 91 cases in which the initial diagnoses were known are shown in Table 3. The wrong initial diagnoses included gastroenteritis, small bowel obstruction, constipation, diverticulitis, appendicitis, and influenza in 27, 13, 10, 5, 3, and 3 cases, respectively.

**Table 3:** List of the wrong initial diagnosis.

List of wrong initial diagnosis (n = 91)	Cases
Gastroenteritis	27
Small bowel obstruction	13
Constipation	10
Diverticulitis	5
Appendicitis	3
Influenza	3
Ischemic colitis	2
Gastrointestinal bleeding	2
Intra-abdominal abscess	2
Inguinal hernia	2
Musculoskeletal disease	2
Disturbance of consciousness	2
Others	18

## Discussion

Lower gastrointestinal perforation is one of the diagnoses of acute abdomen. Unlike in appendicitis and diverticulitis, a myriad of bacteria in the stool spread from the colon to the abdominal cavity, causing acute diffuse peritonitis, bacterial toxin absorption, and infectious shock, leading to multiple organ failure with a high mortality rate of 30–50%^11,13,14^. Therefore, lower gastrointestinal perforation requires prompt diagnosis and therapeutic intervention^11^. However, it has no specific symptoms and is often misdiagnosed. Previous case series have reported that only about 10% of cases are diagnosed before surgery^11^. We conducted the first large-scale multicenter retrospective study on lower gastrointestinal perforation.

In this study, delayed diagnosis occurred in about 31% of the cases; this was less than the rates reported in previous studies^11^. In a previous study, the rate of accurate diagnosis before surgery was approximately 10%^11^; however, in this study, 60–70% of the patients had timely diagnosis. This may be because the prevalence of CT in Japan is extremely high^15^ and the fact that the definition of timely diagnosis included cases in which although the initial diagnosis was wrong, the patients were referred immediately, resulting in timely diagnosis (near miss) and cases in which the immediate diagnosis was difficult (no fault).

There are no previous studies regarding factors associated with diagnostic errors of lower gastrointestinal perforation. Yang and Ni reported that this disease was more common in the elderly and bedridden patients, with 70% of the patients having a history of chronic constipation and 20% developing the disease after laxatives were administered^11^. Therefore, acute abdominal pain with signs of peritoneal irritation in the elderly with chronic constipation or in long-term bedridden patients should be considered as a symptom of lower gastrointestinal perforation^11^. The results of this study indicate that bedridden patients and those with a history of constipation are at risk for lower gastrointestinal perforation; however, these cases were not directly related to delayed diagnosis. Other factors, such as antipsychotic use, analgesic use, use of immunosuppressive drugs including steroids, history of psychiatric disorders, and history of diabetes mellitus, were also unassociated with delayed diagnosis.

In our study, the multivariate logistic regression analysis revealed that examination by practitioners other than general physicians was significantly associated with delayed diagnosis. Furthermore, presence of fever, absence of abdominal tenderness, and unavailability of urgent radiology reports tended to be associated with delayed diagnosis.

In Japan, the training of general practitioners and emergency physicians has only just been established and there is an urgent need to train them further. Currently, most of the primary care and emergency room services are provided by domain-specific specialists^16^. As a result, primary care and emergency medicine in Japan is provided by doctors who are not well trained in these areas. Additionally, the level of accuracy of diagnosis by gastroenterologists and non-gastroenterologists were similar in this study (OR: 1.258, 95% CI: 0.786–2.014). Although this is a problem unique to Japan, the results suggest that training of the general practitioners and emergency physicians who are skilled in dealing with various symptoms decreases the risk of delayed diagnosis of lower gastrointestinal perforations and possibly of other acute illnesses.

The reason that lower gastrointestinal perforations with fever is more likely to be missed is that the presence of fever may anchor the working diagnosis to the occurrence of an infection. With some exceptions, lower gastrointestinal perforation should result in abdominal pain with signs of peritoneal irritation. In the presence of abdominal pain with peritoneal irritation signs, the differential diagnoses include appendicitis, diverticulitis, pancreatitis, or gastrointestinal perforation. Therefore, when patients have abdominal pain with peritoneal irritation signs with or without fever, the strategy to reduce delayed diagnosis is not to treat empirically with antimicrobial agents, but to perform imaging studies to confirm the diagnosis.

This study revealed that the absence of tenderness was associated with delayed diagnosis of lower gastrointestinal perforation, and the finding of an absence of abdominal tenderness in about 10% of the cases is consistent with that in Yang and Ni's study^11^. Lower gastrointestinal perforation without abdominal findings presents a diagnostic difficulty. Repeated reevaluation and follow-up of patients with abdominal pain or positive inflammatory reaction without a clear diagnosis is desirable to avoid missing these cases.

In Japan, the availability of urgent radiology reports is low, with only 26.9% cases reported in this study. Communication barriers between physicians and radiologists are due to a variety of factors, such as system factors (e.g., health information technology, crowding, shift-based work, and interruptions) ^17,18^. Our results suggested that the unavailability of urgent radiology reports may be related to delayed diagnosis. To solve these problems, various factors such as training of the radiologists, improvement of the information technology systems including remote reading, and reform of the medical system are necessary.

The most common initial misdiagnoses in our study cohort were infectious enteritis, small bowel obstruction, constipation, diverticulitis, and appendicitis. In a previous study, upper gastrointestinal perforation was a common diagnosis, while colonic swelling, appendicitis, and pancreatitis were also reported^11^. The tendency for fatal diseases to be overlooked under the diagnosis of gastroenteritis in Japan is consistent with the finding of a previous study by Watari et al. ^19^. It is understandable that constipation is a common initial diagnosis due to the presenting characteristics of the patients. The frequencies of small bowel obstruction, diverticulitis, and appendicitis among initial misdiagnoses are one of the features of this study. If there is small bowel obstruction with fever, increased inflammatory response, and peritoneal irritation signs, it is advisable to review the differential diagnoses. If there is abdominal pain with peritoneal irritation signs, it is advisable to request imaging tests for a definitive diagnosis rather than simply judging the case as diverticulitis or appendicitis and treating it empirically with antimicrobial agents.

The greatest strength of this multicenter Japanese study is its large sample size; however, there are certain limitations to this study. First, the retrospective study design cannot fully exclude several common biases, including information bias, selection bias, and unexpected confounding factors. Second, we did not determine the causes of lower gastrointestinal perforation, because this disease is more common in the elderly and the bedridden, and we presumed many cases to have been managed conservatively or palliatively without surgery. In fact, only about half of the perforation sites could be identified at surgery, and many cases were treated conservatively. Additionally, previous literature has shown that the causes of non-traumatic intestinal perforation are diverse and that it is not necessary to determine them before surgery. Since the main purpose of this study was to determine the accuracy of the initial diagnosis of lower gastrointestinal perforation and because of the abovementioned limitations, causal analysis was excluded from the design. Third, about half of the cases in our study were diagnosed as lower gastrointestinal perforations based on the radiologist's report of the CT images. In about 10% of cases, radiologists reportedly misjudged whether the perforation was in the upper or lower gastrointestinal tract^20^. Although this study excluded cases in which the radiologist could not determine the site of perforation, it should be noted that the radiologist's judgment is not absolute in nonoperative cases. Fourth, since the objective of our study was to mainly identify epidemiological characteristics of delayed diagnosis of lower gastrointestinal perforation, we did not examine the prognosis of the patients. There were many prognostic factors of acute abdomen, and most of the patients with this disease were elderly. Therefore, if we are to accurately analyze whether delayed diagnosis is associated with the prognosis, we must consider the clinical course, detailed data on patient factors and hospital factors, and even palliative cases of perforation due to advanced colorectal cancer. In the future, we hope to conduct a retrospective analysis of error cases wherein the errors might have occurred, to determine what error patterns exist and whether they are preventable or not.

In summary, delayed diagnosis of lower gastrointestinal perforation occurs in about one-third of the cases. Factors associated with delayed diagnosis probably include the presence of fever, absence of abdominal tenderness, unavailability of urgent radiology reports, and examination by a non-generalist.

## Data Availability

The datasets generated during and/or analyzed during the current study are available from the corresponding author on reasonable request.

## References

[1] Graber ML (2020). Progress understanding diagnosis and diagnostic errors: Thoughts at year 10. Diagnosis (Berl).

[2] Singh H, Meyer AN, Thomas EJ (2014). The frequency of diagnostic errors in outpatient care: Estimations from three large observational studies involving US adult populations. BMJ. Qual. Saf..

[3] Shojania KG, Burton EC, McDonald KM, Goldman L (2003). Changes in rates of autopsy-detected diagnostic errors over time: A systematic review. JAMA.

[4] Thomas EJ, Studdert DM, Burstin HR, Orav EJ, Zeena T, Williams EJ, Howard KM, Weiler PC, Brennan TA (2000). Incidence and types of adverse events and negligent care in Utah and Colorado. Med. Care.

[5] Brennan TA, Leape LL, Laird NM, Hebert L, Localio AR, Lawthers AG, Newhouse JP, Weiler PC, Hiatt HH (1991). Incidence of adverse events and negligence in hospitalized patients. Results of the Harvard Medical Practice Study I. N. Engl. J. Med..

[6] Tehrani ASS, Lee H, Mathews SC, Shore A, Makary MA, Pronovost PJ, Newman-Toker DE (2013). 25-Year summary of US malpractice claims for diagnostic errors 1986–2010: An analysis from the national practitioner data bank. BMJ. Qual. Saf..

[7] Balogh E, Miller B, Ball J (2015). Improving Diagnosis in Health Care.

[8] Economic Cycle Research Institute. Top 10 patient safety concerns 2020. (2020). https://www.ecri.org2020.9, Accessed 08 May 2021.

[9] Lanas A, García-Rodríguez LA, Polo-Tomás M, Ponce M, Alonso-Abreu I, Perez-Aisa MA, Perez-Gisbert J, Bujanda L, Castro M, Muñoz M, Rodrigo L, Calvet X, Del-Pino D, Garcia S (2009). Time trends and impact of upper and lower gastrointestinal bleeding and perforation in clinical practice. Am. J. Gastroenterol..

[10] Brown CV (2014). Small bowel and colon perforation. Surg. Clin. North Am..

[11] Yang B, Ni HK (2008). Diagnosis and treatment of spontaneous colonic perforation: Analysis of 10 cases. World J. Gastroenterol..

[12] Kanda Y (2013). Investigation of the freely available easy-to-use software 'EZR' for medical statistics. Bone Marrow Transplant..

[13] Kasahara Y, Matsumoto H, Umemura H, Shirafa S, Kuyama T (1981). Idiopathic perforation of the sigmoid colon in Japan. World J. Surg..

[14] Serpell JW, Nicholls RJ (1990). Stercoral perforation of the colon. Br. J. Surg..

[15] OECD. Computed tomography (CT) scanners. https://data.oecd.org/healtheqt/computed-tomography-ct-scanners.htm. Accessed 30 June 2021(2021).

[16] Higashi H, Takaku R, Yamaoka A, Lefor AK, Shiga T (2019). The dedicated emergency physician model of emergency care is associated with reduced pre-hospital transportation time: A retrospective study with a nationwide database in Japan. PLoS ONE.

[17] Fairbanks RJ, Guarrera TK, Bisantz AB, Venturino M, Westesson PL (2010). Opportunities in IT support of workflow & information flow in the emergency department digital imaging process. Proc. Hum. Factors Ergon. Soc. Ann. Meet..

[18] Enayati M, Sir M, Zhang X, Parker SJ, Duffy E, Singh H, Mahajan P, Pasupathy KS (2021). Monitoring diagnostic safety risks in emergency departments: Protocol for a machine learning study. JMIR. Res. Protoc..

[19] Watari T, Tokuda Y, Mitsuhashi S, Otuki K, Kono K, Nagai N, Onigata K, Kanda H (2020). Factors and impact of physicians' diagnostic errors in malpractice claims in Japan. PLoS ONE.

[20] Drakopoulos D, Arcon J, Freitag P, El-Ashmawy M, Lourens S, Beldi G, Obmann VC, Ebner L, Huber AT, Christe A (2021). Correlation of gastrointestinal perforation location and amount of free air and ascites on CT imaging. Abdomen Radiol. (NY).

